# SPIC: A novel similarity metric for comparing transcription factor binding site motifs based on information contents

**DOI:** 10.1186/1752-0509-7-S2-S14

**Published:** 2013-12-17

**Authors:** Shaoqiang Zhang, Xiguo Zhou, Chuanbin Du, Zhengchang Su

**Affiliations:** 1 College of Computer Science and Information Engineering, Tianjin Normal University, Tianjin, 300387, China; 2 Department of Bioinformatics and Genomics, Bioinformatics Research Center, the University of North Carolina at Charlotte, NC 28223, USA

**Keywords:** gene regulatory networks, information contents, transcription factor binding site (TFBS), motif, similarity metric

## Abstract

**Background:**

Discovering transcription factor binding sites (TFBS) is one of primary challenges to decipher complex gene regulatory networks encrypted in a genome. A set of short DNA sequences identified by a transcription factor (TF) is known as a motif, which can be expressed accurately in matrix form such as a position-specific scoring matrix (PSSM) and a position frequency matrix. Very frequently, we need to query a motif in a database of motifs by seeking its similar motifs, merge similar TFBS motifs possibly identified by the same TF, separate irrelevant motifs, or filter out spurious motifs. Therefore, a novel metric is required to seize slight differences between irrelevant motifs and highlight the similarity between motifs of the same group in all these applications. While there are already several metrics for motif similarity proposed before, their performance is still far from satisfactory for these applications.

**Methods:**

A novel metric has been proposed in this paper with name as SPIC (Similarity with Position Information Contents) for measuring the similarity between a column of a motif and a column of another motif. When defining this similarity score, we consider the likelihood that the column of the first motif's PFM can be produced by the column of the second motif's PSSM, and multiply the likelihood by the information content of the column of the second motif's PSSM, and vise versa. We evaluated the performance of SPIC combined with a local or a global alignment method having a function for affine gap penalty, for computing the similarity between two motifs. We also compared SPIC with seven existing state-of-the-arts metrics for their capability of clustering motifs from the same group and retrieving motifs from a database on three datasets.

**Results:**

When used jointly with the Smith-Waterman local alignment method with an affine gap penalty function (gap open penalty is equal to1, gap extension penalty is equal to 0.5), SPIC outperforms the seven existing state-of-the-art motif similarity metrics combined with their best alignments for matching motifs in database searches, and clustering the same TF's sub-motifs or distinguishing relevant ones from a miscellaneous group of motifs.

**Conclusions:**

We have developed a novel motif similarity metric that can more accurately match motifs in database searches, and more effectively cluster similar motifs and differentiate irrelevant motifs than do the other seven metrics we are aware of.

## Background

As one of the most important cellular functions, transcriptional regulation determines the specific gene products in a cell, upon which all the other cellular functions are based [1,2]. Transcriptional regulation is triggered by the binding of TF proteins to 6-25 bps (base pairs) specific DNA sequences called *cis*-regulatory elements (CREs) or transcription factor binding sites (TFBSs) in a gene's promoter region or remote regulatory regions such as enhancers, silencers and insulators [3]. These TF-DNA interactions in a cell form the transcriptional regulatory network (TRN) of the cell [4]. In principle, TRNs of all cell types of an organism are encoded in its genome, however, deciphering these TRNs from the genome sequence turns out to be one of a very challenging tasks [5,6]. The first step to this goal is to recognize all TFBSs in a genome [5,7,8]. Although the binding sites of the same TF usually have a certain conservative feature and the same length, they can show some level of degeneration, and be located in very long non-coding sequences, making their computational prediction very difficult [9]. A set of the same TF's conserved binding sites is always called a *motif*, which can be verified by experiments or predicted by comparing a set of DNA sequences potentially containing the TFBSs. A lot of *de novo *motif-finding algorithms have been developed to identify TFBSs because they are often more conserved than their surrounding DNA segments [9]. A position frequency matrix (PFM) or a position-specific scoring matrix (PSSM) is always employed to represent a motif [9,10]. The two matrices are deformed from the alignments of its individual binding site sequences, and largely mirror the position binding preference of the corresponding TF. Thus, we can use one of the two matrices to scan the sequences potentially containing TFBSs to discover them [10].

After using motif finding tools to get some putative motifs, we often want to infer the TFs affiliated to them by looking for their matching motifs in a validated TFBS motif database [11], or to cluster similar sub-motifs of the same TF obtained by different methods to remove redundancies or to form a complete motif [11-13]. Moreover, the motifs of a TF family also show some level of similarity to form a familial binding profile (FBP) because these TFs in a family belong to a structurally related class [14,15]. Consequently, an efficient metric is desired for measuring the motif-motif similarity in the applications mentioned above. Most of current motif comparison methods are divided into two parts: a column similarity metric for comparing two columns which come from the PFMs (or the PSSMs) of two motifs respectively, and a pairwise alignment algorithm for the two motifs using the column similarity metric and a penalty function for gaps [11]. The metrics to measure column-to-column motif similarity mainly include sum of squared distances (SSD) [15,16], *p*-value of Chi-square (pCS) [17], average log-likelihood ratio (ALLR) [18], average Kullback-Leibler (AKL) [19], Pearson's correlation coefficient (PCC) [20]. Either the Needleman-Wunsch [21] or the Smith-Waterman [22] algorithms used to be applied to search for the optimal alignment assuming an affine gap penalty function. Mahony *et al*. have built a web server STAMP which integrated these metrics and alignment algorithms after assessing them [11,23]. Besides these metrics along with alignment algorithms, two alignment-free metrics for comparing motifs, Mosta and KFV, were designed by Pape *et al*. [24] and by Xu and Su [25], respectively. The two alignment-free metrics and these in STAMP have been evaluated by Xu and Su [25], in which the KFV method was showed to be better than Mosta and the others.

Note that the seven metrics mentioned above only employed PFMs. None of them uses the column information contents (ICs) and PSSMs. In fact, if the total ICs of two motifs are low, they may have high similarity score due to high correlation between each pair of columns. So if two motifs have columns with low ICs, we need to delete these low IC columns before using these metrics for the comparison. These metrics work well to cluster similar motifs but can hardly separate true motifs from spurious ones with low IC columns.

Here we presented a novel metric named SPIC (Similarity with Position Information Contents) with better performance for column-to-column motif comparison. In our genome-wide TFBS motif prediction tools GLECLUBS [12] and eGLECLUBS [13] for prokaryotes through comparative genomics, a similar metric with ungapped alignment has been proposed. In this paper, we improved the metric by considering the different alignment algorithms with gap functions. Especially, besides the PFMs and PSSMs, the information content of each position was involved into the SPIC metric. More specifically, for any two columns separately from two motifs, SPIC first computes a score between the PSSM multiplied by the IC of one column and the PFM of the other column, and vice versa. The similarity between the two columns is then defined based on the results with normalization. When evaluated on the datasets from STAMP [26], KFV [25], and GLECLUBS [12,13], SPIC outperforms all the existing metrics for recovering motifs by searching a database and grouping closely related motifs.

## Methods

### Previous metrics

The STAMP tool contains five column similarity metrics. The detail definitions of these metrics are summarized in Table 1. In these definitions, for each column X  of a PFM, $X_{b}$ denotes the probability of each base b , $\bar{X}$ the average of $X_{b}$, $N_{X}$ the total counts of all bases, and $N_{X_{b}}$ the total counts of base b . $N_{X_{b}}^{e}=\left(N_{X}\cdot N_{X_{b}}\right)/N$. $q_{b}$ denotes the background probability of each base b  and is assumed to be 0.25 for all bases. In the Asymptotic Covariance (AC) metric designed by Pape *et al*. [24], the asymptotic covariance between the counts N(m) of all binding sites separately from two TFBS motifs and their reverse complementary TFBSs in a *m*-length background sequence is calculated (see Table 1). The KFV (*k*-mer frequency vector) metric, recently proposed by Xu and Su [25], first converts each PFM of length *k *into a $4^{k}$-dimension composition vector and then use cosine angle to calculate the similarity between the vectors of two motifs.

**Table 1:** The definitions of six metrics used for motif comparison.

Similarity metric	Formula	References
Average log-likelihood ratio (ALLR)	$ALLR\left(X,Y\right)=\frac{\underset{b}{\sum }N_{X_{b}}\left(\frac{Y_{b}}{q_{b}}\right)+\underset{b}{\sum }N_{Y_{b}}\left(\frac{X_{b}}{q_{b}}\right)}{\underset{b}{\sum }\left(N_{X_{b}}+N_{Y_{b}}\right)}$	Wang and Stormo [18]

Average Kullback-Leibler *(*AKL*)*	$AKL\left(X,Y\right)=10-\frac{\underset{b}{\sum }X_{b}\log\frac{X_{b}}{Y_{b}}+\underset{b}{\sum }Y_{b}\log\frac{Y_{b}}{X_{b}}}{2}$	Kullback and Leibler [19]

Sum of squared distances (SSD)	$SSD\left(X,Y\right)=2-\left(\underset{b}{\sum }{\left(X_{b}-Y_{b}\right)}^{2}\right)$	Schones *et al*. [17]

1-p-value of Chi-square (pCS)	$\chi^{2}\left(X,Y\right)=\underset{b}{\sum }\frac{{\left(N_{X_{b}}-N_{X_{b}}^{e}\right)}^{2}}{N_{X_{b}}^{e}}+\underset{b}{\sum }\frac{{\left(N_{Y_{b}}-N_{Y_{b}}^{e}\right)}^{2}}{N_{Y_{b}}^{e}}$	Schones *et al*. [17]

Pearson correlation coefficient *(*PCC*)*	$PCC\left(X,Y\right)=\frac{\underset{b}{\sum }\left(X_{b}-\bar{X}\right)\left(Y_{b}-\bar{Y}\right)}{\sqrt{\underset{b}{\sum }{\left(X_{b}-\bar{X}\right)}^{2}\underset{b}{\sum }{\left(Y_{b}-\bar{Y}\right)}^{2}}}$	Pietrokovski [20]

Asymptotic Covariance (AC)	$AC\left(A,B\right)=\underset{m\to \infty }{\lim}m^{-1}\text{cov}\;\left(N_{A}\left(m\right)+N_{A^{\prime }}\left(m\right),N_{B}\left(m\right)+N_{B^{\prime }}\left(m\right)\right)$	Pape *et al*. [24]

### The SPIC Metric

Given a motif $M_{i}$ composed of $n_{i}$ TFBSs with a length $L_{i}$, let $F_{i}={\left(f_{i}\left(b,X\right)\right)}_{4\times L_{i}}$ be its PFM and $P_{i}$ be its PSSM defined as,

(1) $$P_{i}={\left(P_{i}\left(b,X\right)\right)}_{4\times L_{i}}={\left(\log\frac{p_{i}\left(b,X\right)}{q_{i}\left(b\right)}\right)}_{4\times L_{i}},$$

where $q_{i}\left(b\right)$ denotes the probability of base b  contained in background sequences, $p_{i}\left(b,X\right)$ and $f_{i}\left(b,X\right)$ are the probability and number of base b  located at the column X  of $P_{i}$, respectively. Note that a pseudo-count is required for calculating these probabilities. The definition of the information content (IC) of column X  is as below,

(2) $$I\left(X,P_{i}\right)=\underset{b}{\sum }p_{i}\left(b,X\right)P_{i}\left(b,X\right)=\underset{b}{\sum }p_{i}\left(b,X\right)\log\frac{p_{i}\left(b,X\right)}{q_{i}\left(b\right)}.$$

Given two PFMs *F*_1 _and *F*_2 _and two PSSMs *P*_1 _and *P*_2 _of two motifs $M_{1}$ and $M_{2}$ respectively, the similarity value between two columns X  and Y  from $M_{1}$ and $M_{2}$ respectively is computed by

(3) $$\text{Sim}\left(M_{1}\text{(}X\text{),}M_{2}\left(Y\right)\right)=\min\left\{1,\frac{\max\left\{\text{S}\left(P_{1}\left(X\right),F_{2}\left(Y\right)\right),\text{S}\left(P_{2}\left(Y\right),F_{1}\left(X\right)\right)\right\}}{\max\left\{\text{S}\left(P_{1}\left(X\right),F_{1}\left(X\right)\right),\text{S}\left(P_{2}\left(Y\right),F_{2}\left(Y\right)\right)\right\}}\right\},$$

where

(4) $$\text{S}\left(P_{i}\left(A\right),F_{j}\left(B\right)\right)=I\left(A,P_{i}\right)\underset{b}{\sum }\left(f_{j}\left(b,B\right)\cdot \log\frac{p_{i}\left(b,A\right)}{q_{i}\left(b\right)}\right).$$

In the formula (4), the column ICs are used to enhance the effect of the columns of a motif with high information and weaken the influence of the columns with low information on the similarity score. It must be noted that the formula (4) indicates the likelihood of *P_i_*(*A*) generating *F_j_*(*B*). The denominator used to normalize the scores in the similarity function (3) is generally the upper bound of the numerator. In rare instances, the numerator in function (3) may be greater than the denominator, so the number "1" is also used to normalize the scores.

### Pairwise column alignment

To compute the similarity between two motifs, we first need to make an alignment between their columns. We consider both local and global alignments between two motifs that are similarly defined as in the pair-wise sequence alignments [11]. Let Ω(*M*_1_(*X*), *M*_2_(*Y*), *G*) be any alignment between two motifs *M*_1 _and *M*_2 _with gaps *G*, where column *X *of *M*_1 _is aligned with column *Y *of *M*_2_. The similarity score between motifs *M*_1 _and *M*_2 _with the alignment is defined as,

(5) $$\text{S}\left(M_{1}\text{,}M_{2},\Omega \right)=\underset{\begin{array}{c} \text{all aligned }\\ \text{pairs}\left(X,Y\right) \end{array}}{\sum }\text{Sim}\left(M_{1}\text{(}X\text{),}M_{2}\left(Y\right)\right)-g\left(G\right),$$

where $\text{Sim}\left(M_{1}\text{(}X\text{),}M_{2}\left(Y\right)\right)$ is the similarity between the two aligned columns *M*_1_(*X*) and *M*_2_(*Y*) and computed by a column similarity metric, and *g*(*G*) is a gap penalty function. So the motif-motif similarity score is defined as the score of the best alignment between motifs *M*_1 _and *M*_2_, i.e.,

(6) $$\text{Sim}\left(M_{1}\text{,}M_{2}\right)=\underset{\Omega }{\max}\text{Sim}\left(M_{1}\text{,}M_{2},\Omega \right).$$

For a given column similarity metric, we compute the similarity score between two motifs using the Needleman-Wunsch (NW) global alignment algorithm [21] or the Smith-Waterman (SW) local alignment algorithm [22], assuming an affine gap penalty function with the gap-extension penalty being half of the gap-opening penalty. An extended SW alignment algorithm without gaps is also evaluated. Furthermore, an empirical *p*-value is assigned to the similarity score to measure the likelihood between two aligned motifs [15].

### Datasets of motifs

In this study three dataset of motifs verified by experiments are employed for testing and evaluation purpose. Dataset-1, first chosen from JASPAR by *Mahony et al*. [11], is composed of 96 true motifs which belong to 13 known TF structural classes. Among these motifs, 25 motifs belong to the Zinc-Finger (ZF) family. Dataset-2, created by Xu and Su [25] for testing the outstanding ability of the KFV metric to identify redundant PFMs, is composed of 124 JASPAR core motifs and three sub-motifs for each core motif by randomly selecting its two-thirds of sequences. Dataset-3, available at: http://gleclubs.uncc.edu/pbs, contains about 10^5 ^putative motifs that were predicted in our earlier work [12,13] from more than two thousand sets of genome-wide orthologous intergenetic sequences in *E. coli *K12 and other 54 reference genomes of gamma-proteobacteria. Referred to the database RegulonDB (version 6) [27], these predicted motifs cover 1,411 known TFBSs of 122 true motifs (or TFs) in *E. coli *K12. More details of the three datasets are summarized in Table 2.

**Table 2:** Summary of the three datasets used for the evaluation in this study.

	Number of true motifs	Number of putative motifs	Number of classes	Average length	True motifs source	Data source
Dataset-1	96	0	13	10.39	JASPAR	Mahony, *et al*., 2007 [11]

Dataset-2	124	0	Unknown	10.6	JASPAR	Xu and Su, 2010 [25]

Dataset-3	122	$10^{5}$	Unknown	16	RegulonDB	Zhang, *et al*., 2009 [12]

### Implementation of metrics

The seven metrics (PCC, AKL, ALLR, pCS, SSD, AC, and KFV) listed in Table 1 were employed to compare with SPIC for their ability to cluster relevant true motifs, filter out fake motifs, or recover motifs from a database. We used the STAMP platform for computing the first five alignment-dependent metrics scores http://www.benoslab.pitt.edu/stamp/, the Mosta package included in SABINE for computing the AC scores http://www.ra.cs.uni-tuebingen.de/software/SABINE/downloads/index.htm, and the web server of KFV for computing the KFV scores http://bioinfo.uncc.edu/kfv/.

### Performance assessing

In order to inspect the ability of these metrics to recognize the motifs of the same TFs in Dataset-1 and Dataset-2, the ROC (Receiver Operating Characteristic) curves were plotted. In database searches, we define the "performance accuracy" as the percent of motifs correctly recovered by using the best-hit method. The ROC profiles were drawn based on the rule described below. Given a dataset consisting of *n *motifs whose TF structural classes are known, we list all of *n*(*n*+1)/2 pairs of motifs and compute the similarity scores of each pair using SPIC and the other metrics. We set two motifs as a mismatch if the similarity score between them is less than a threshold or a match, otherwise. We call a match a true positive (TP) if the two motifs belong to the same FBP , and a mismatch a true negative (TN) if the two motifs belong to different FBPs. The ROC curve is represented by the TP rate against the FP rate under different motif similarity thresholds.

## Results and discussions

### Motif retrieval

Given the profile of a motif whose cognate TF information is unknown, one of frequently used applications is to search the motif in a database. A column similarity metric associated with an alignment algorithm or an alignment-free similarity metric is employed to compare the query motif to each motif in the database. The motifs are "hit" by the query motif if their similarity score are over a threshold in the database [11]. However, the motifs of TFs either belonging to the same TF family or in a closely evolutionary relationship show some degree of similarity while the binding sites in a motif sometimes show highly degenerate. So it is often difficult to distinguish similar motifs and identify the required motifs precisely in a database. The SSD, PCC and KFV metrics are chosen for the comparison with SPIC for their capability of retrieving motifs of a same TF family in Dataset-1. It is because that SSD, PCC and KFV were shown to have the better performance than the other three column similarity metrics joint with an optimal alignment [11] and the alignment-free AC score [25]. As described in Xu and Su [25], the accuracy of a metric is calculated as the percent of motifs whose TF families are "best hit" by the metric in a dataset of motifs.

As evaluated by Mahony *et al*. [11], the PCC metric combined with the SW ungapped alignment algorithm (PCC/SWU), and the SSD metric combined with SW alignment (SSD/SW) with gap extension equal to 0.5 and gap open equal to 1, are the best two metric and alignment settings on Dataset-1 among the five column similarity metrics associated with their all possible alignment settings. According to Xu and Su [25], when 4-mer and cosine angle are used for vector construction and comparison, the KFV results in the best results. Here we also used the NW and SW alignment algorithms respectively to test the SPIC with almost all of different gap open penalties (gap extension is always set as half the gap open). The top seven performing alignment strategies of SPIC and the optimal strategies of PCC/SWU, SSD/SW and KFV, are listed in Table 3. Among these strategies, the combination of the SPIC metric and the Smith-Waterman algorithm (SPIC/SW) with gap open equal to 1 achieves the highest accuracy on Dataset-1. The results in Table 3 show that SPIC has more superior strategies than the other metrics.

**Table 3:** Comparison of top 7 performing alignment strategies of SPIC with the best strategies of existing methods for motif retrieval on Dataset-1.

	Accuracy		
	
Strategy	ZF PFMs(25)	Non-ZF PFMs(71)	Total(96)
SPIC/SW(gap open = 1.00)	**0.620**	**0.921**	**0.841**
SPIC/SW(gap open = 0.75)	0.613	0.918	0.837
SPIC/SW(gap open = 0.50)	0.614	0.916	0.837
SPIC/SW(gap open = 1.50)	0.605	0.916	0.835
SPIC/SW(gap open = 0.25)	0.606	0.915	0.835
SPIC/SW(ungapped)	0.610	0.916	0.836
SPIC/NW(gap open = 1.0)	0.585	0.793	0.731
KFV(4-mer, cosine angle)	0.600	0.915	0.833
PCC/SWU	0.600	0.887	0.813
SSD/SW	0.560	0.859	0.781

The last three columns are the results for the zinc-finger (ZF), non-ZF, and total families, respectively. The performances of SSD/SW and PCC/SWU are quoted from the STAMP [11]. The data of KFV are quoted from [25]. Gap extension is equal to half the gap open.

**Figure 1:**
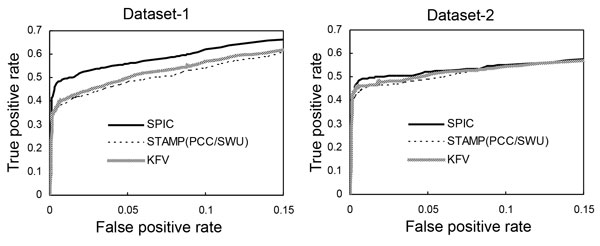
**Evaluation of the three motif similarity metrics using ROC analysis on Dataset-1 and Dataset-2**.

For further comparison of our best strategy SPIC/SW (gap open = 1) with the strategy PCC/SWU which has the best performance in STAMP and the optimal strategy of KFV (4-mer, cosine angle) for recovering motifs from a dataset, we do ROC analysis of the three strategies' performance on Dataset-1 and Dataset-2. As exhibited in Figure 1, SPIC/SW (gap open = 1) performs more outstandingly than the two strategies PCC/SWU in STAMP and KFV (4-mer, cosine angle) for motif recovery on Dataset-1 and Dataset-2.

### Separation of true motifs from spurious motifs

In some algorithms for genome-wide prediction of transcription factor binding sites based on phylogenetic footprinting such as GLECLUBS [12,13] and PhyloNet [16], sub-motifs and redundant motifs of any TF are required to be merged together into a unique motif, meanwhile, spurious motifs are required to be discarded [12,13,16]. To this end, we desire to get a metric that not only precisely measures the pairwise motif similarity, but also effectively differentiates irrelevant motifs. More specifically, the desired metric can assign a similarity score high enough for two sub-motifs of the same TF motif, and a similarity score low enough for two motifs without any evolutionary relationship to separate true motifs from spurious ones. Dataset-3 generated by GLECLUBS [12,13] is composed of massive amounts of spurious motifs and a tiny fraction of true motifs. In order to discover true motifs from Dataset-3, we need to evaluate the SPIC and the other seven metrics for their ability to cluster sub-motifs of each TF into a motif and separate true motifs from spurious ones.

**Figure 2:**
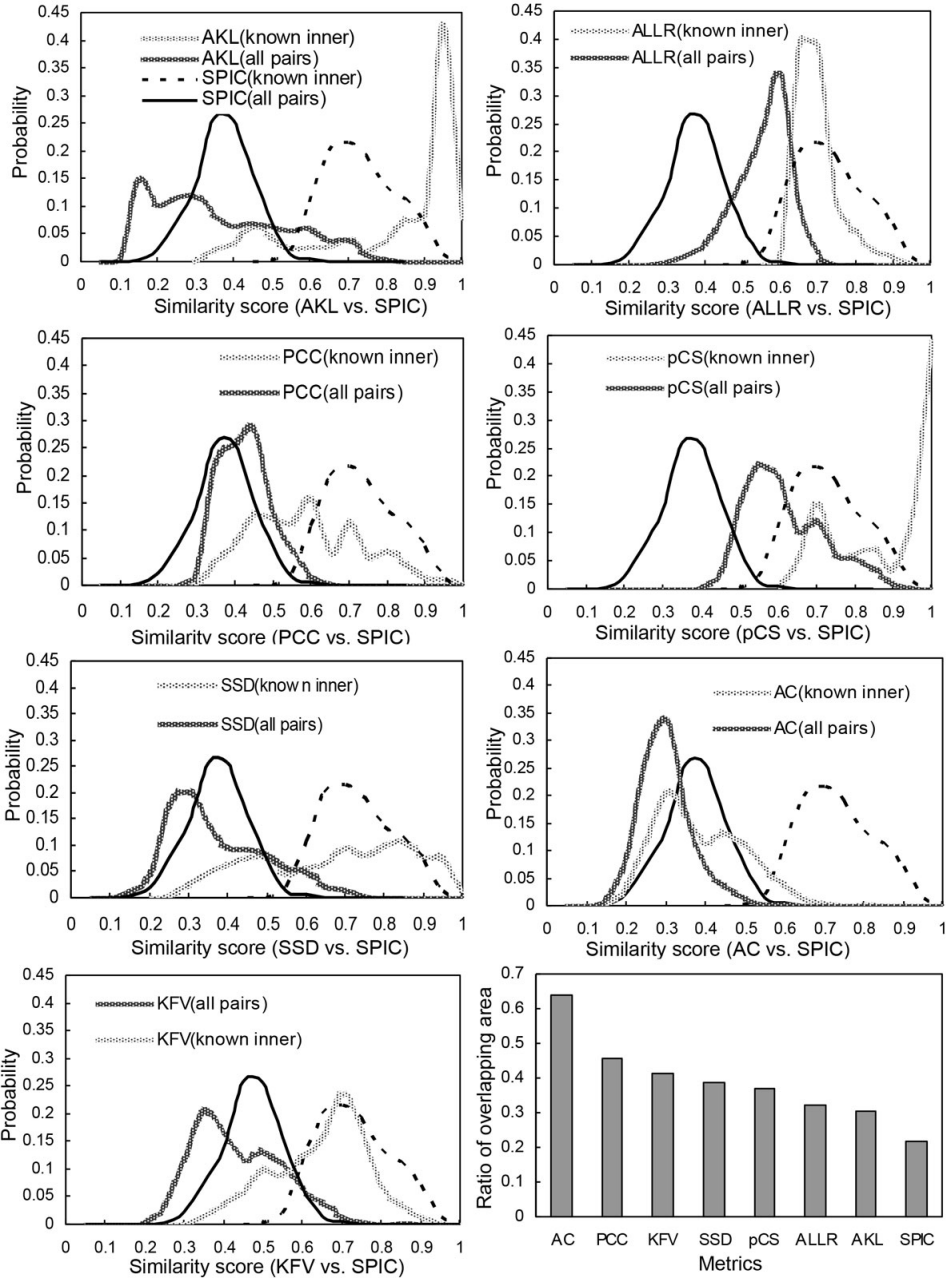
**Comparison of SPIC with the seven existing methods for separation of relevant motifs from irrelevant ones on Dataset-3**.

For this purpose, we need a group of true motifs used for evaluation on Dataset-3. 122 TF motifs of *E. coli *K12 in ReglonDB are picked out to generate plenty of sub-motifs. For each TF motif consisting of *n *BSs (n≥3), we randomly split it into a sub-motif of size *k *and a sub-motif of size n-k for each k∈{1,2,...,[n/2]}. So [n/2] pairs of sub-motifs can be generated for a motif of size *n*. For each sub-motif of size *k*, we repeat the foregoing split procedure on each sub-motif to generate [k/2] pairs of sub-sub-motifs (also called sub-motifs afterwards). The procedure can be terminated when the size of each sub-motif is 1. We then employ these metrics with their best strategies to calculate the corresponding similarity scores between each pair of sub-motifs [11,25] as well as the scores between each pair of motifs in Dataset-3. As shown in Figure 2, the curves labeled by "all pairs" are the distributions of the similarity scores between each pair of motifs in Dataset-3 after score normalization, and the curves labeled by "known inner" are the distributions of the normalized similarity scores between each pairs of true sub-motifs. Due to the relevance between each pair of true sub-motifs and the irrelevance among most of the motifs in Dataset-3, a metric with outstanding performance should depart the curve labeled by "all pairs" from that labeled by "known inner" very well. As shown in the charts of Figure 2, comparing the two curves generated by SPIC with these by other metrics, we find that the two areas under SPIC's distribution curves have the smallest overlap. Specially, the last chart of Figure 2 collects their overlapping rates which demonstrate that SPIC has the highest performance among these existing metrics in recovering true motifs and separating them from spurious ones.

## Conclusions

Because many applications contain the motif comparison procedure, we proposed a novel similarity metric SPIC based on column information contents. When used jointly with the SW alignment algorithm, it achieves a better performance than the best strategies of those existing metrics in recovering motifs in a database, grouping relevant motifs, merging sub-motifs or redundant motifs, or digging true motifs out of chaos.

## Availability

The C++ program of SPIC including an example can be downloaded freely from our home pages: http://bioinfo.uncc.edu/szhang or http://it.tjnu.edu.cn/sqzhang.

## Competing interests

The authors declare that they have no competing interests.

## Authors' contributions

SZ and ZS conceived the project. SZ and CD designed the metric. SZ and XZ implemented and conducted the experiments. ZS and SZ wrote the paper.

## Declarations

The publication of this article has been funded by a grant (61103073, SZ) from National Science Foundation of China, a grant (11JCYBJC26600, SZ) from Natural Science Funds of Tianjin, a grant from Doctoral Funds of Tianjin Normal University (52X09013, LJ), and two grants (EF0849615 and CCF1048261, ZS) from National Science Foundation of USA.

This article has been published as part of *BMC Systems Biology *Volume 7 Supplement 2, 2013: Selected articles from The 6^th ^International Conference of Computational Biology. The full contents of the supplement are available online at http://www.biomedcentral.com/bmcsystbiol/supplements/7/S2.
